# Quality Assessment of Tomato Paste Products on the Ghanaian Market: An Insight Into Their Possible Adulteration

**DOI:** 10.1155/2024/8285434

**Published:** 2024-09-09

**Authors:** Abena Boakye, Doreen D. Avor, Isaac K. Amponsah, William O. Appaw, Leslie Owusu-Ansah, Silas Adjei, Michael K. Baah, John N. Addotey

**Affiliations:** ^1^ Department of Food Science and Technology College of Science Kwame Nkrumah University of Science and Technology, Kumasi, Ghana; ^2^ Department of Pharmacognosy Faculty of Pharmacy and Pharmaceutical Sciences Kwame Nkrumah University of Science and Technology, Kumasi, Ghana; ^3^ Department of Herbal Medicine Faculty of Pharmacy and Pharmaceutical Sciences Kwame Nkrumah University of Science and Technology, Kumasi, Ghana; ^4^ Mycotoxin and Food analysis Laboratories Department of Food Science and Technology College of Science KNUST, Kumasi, Ghana; ^5^ Food Laboratory Department Food and Drugs Authority, Accra, Ghana; ^6^ Department of Pharmaceutical Chemistry Faculty of Pharmacy and Pharmaceutical Sciences Kwame Nkrumah University of Science and Technology, Kumasi, Ghana

**Keywords:** adulteration, artificial colourant, erythrosine, starch content, titratable acidity, tomato concentrates

## Abstract

Tomato paste is the most consumed tomato product on the Ghanaian market, the majority of which are imported into the country. This food product is easily adulterated, and thus, routine quality checks are necessary. Therefore, the current study is aimed at assessing the quality of eight tomato paste products on the Ghanaian market and checking for the presence of starch and artificial colourant erythrosine as possible adulterants. Routine quality metrics such as the pH, titratable acidity, total solids, and total soluble solids were assessed using standard methods. An HPLC method was employed to detect the presence of the colourant erythrosine, whereas starch content was determined by an enzymatic method using *α*-amylase and then amyloglucosidase. Fifty percent of the products did not qualify to be called tomato paste based on total solid estimation. All the sampled products contained some amount of starch, with three having more than 10 g/100 g of this thickener. Additionally, the banned colourant erythrosine was detected in two of the products. All other parameters were consistent with regulatory standards. The present study has shown that some tomato paste products on the Ghanaian market contain additives that are not permitted under any circumstance and fall short of regulatory standards.

## 1. Introduction

Food adulteration is the process of reducing the quality of food by the addition of foreign materials or the removal of a vital ingredient to maximize profit [1, 2]. Two types of adulteration exist: intentional adulteration, which occurs due to deliberate actions by the producer, and accidental adulteration, which could arise from a lack of adherence to good practices in production [1]. Intentionally adding foreign materials/ingredients is the most common form of adulteration in processed foods. Although additives are accepted and often required in processed foods, each food product has its allowable additives and specified limits [2] to safeguard consumers' health and product quality. Therefore, using external unsanctioned ingredients is an unacceptable practice considering the associated health risks and unethical economic gains of manufacturers at consumers' expense [3].

Global efforts to curb such practices have resulted in many investigations cataloguing specific cases of adulteration, their potential health effects, and economic losses to nations. Choudhary et al. [4] reported synthetic chemicals and detergent powders in tea and cereals, which caused irreparable damage to Indian society. Others have reported adulterants such as foreign leaves or exhausted tea leaves and artificially coloured sawdust in tea and sand or stones in cereals [5]. Honey and oils are adulterated with a range of products, including sugar syrups and molasses in honey and white oils and petroleum fractions in edible oils [5]. The Ghanaian market is not exempted from this menace, with reported cases on the use of Sudan IV dye in palm oil [6] and adulterations of packaged flour products with sawdust and sandpaper. Generally, perpetrators of such illicit acts target popular convenience (ready-to-use) products and/or acclaimed health-beneficial products on local and international markets.

A universally consumed commodity with a high potential for adulteration but which has received less research attention is commercial tomato paste and mix, the classification of which depends on the absence or addition of additives and the percentage of total soluble solids (TSSs) [7]. Tomato paste, the most popular and preferred tomato product, should have not less than 24% of TSSs with no additives [8]. It is currently a household commodity, popular among all classes of consumers in Ghana, as it is acclaimed for its versatile end use, nutritional value, and convenience. A growing public concern is the influx of dozens of new and cheap brands into local markets amidst news reports [9] and speculations of widespread adulterations of the products. However, scientific data is lacking to aid consumers and local authorities in taking evidence-based measures and decisions.

**Figure 1:**
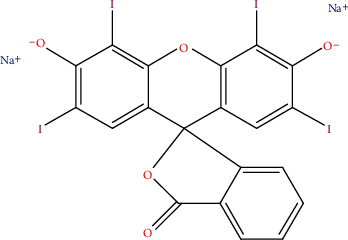
Structure of erythrosine.

Primary possible adulterants for tomato paste are cheap flours (starches) and artificial colours, the most dangerous being a cancer-causing dye, erythrosine [10]. Erythrosine, a poly-iodinated xanthene red dye (Figure 1) used in food products, is reported to induce endotoxic and mutagenic effects in HepG2 cells [11], impairs thyroid function [12], and inhibits drug-metabolizing enzymes [13]. It caused hyperthyroidism and thyroid cancer in rodents. Studies have linked erythrosine consumption to altered cognition and behaviour in children [14]. Elsewhere, its consumption is linked to an increase in the incidence of thyroid tumours through the induction of chromosome aberrations [15, 16].

The use of the dye in food products in Ghana is banned [17, 18], and this is also the case in the countries Norway and Spain [19]. Therefore, the current study is aimed at conducting a market inventory of tomato paste products in the two largest cities of Ghana and assessing their quality per recommended product specifications, including the presence of starch and artificial colourant erythrosine as possible adulterants.

## 2. Materials and Methods

### 2.1. Materials and Reagents

All reagents were of analytical grade. Reference erythrosine dye, methanol, ethanol, N/50 iodine, sodium acetate buffer, ammonium acetate buffer, and acetonitrile were obtained from Merck (Darmstadt, Germany). Amyloglucosidase, *α*-amylase, and D-glucose were obtained from Sigma (St. Louis, MO, United States). Glucose oxidase, peroxidase, and 4-aminoantipyrine (GOPOD) reagent were obtained from Megazyme (Bray, Ireland). Tomato paste samples were obtained from the selected markets.

### 2.2. Study Area and Design

n inventory of available tomato paste brands was conducted at various markets in the two major cities in Ghana: Accra and Kumasi. Markets were selected based on two factors: (1) the importance to trade in the subregion and (2) the importance to trade in Ghana. A smaller localised market was used where it was challenging to obtain different sample batches. Selected markets were Makola market in Accra and Asafo and “Central” markets in Kumasi, with Ayeduase in Kumasi being the smaller market. All instrumental tests were conducted in laboratories of the Department of Pharmacognosy, Food Science and Technology, KNUST, and the Food and Drugs Authority (FDA), Ghana.

### 2.3. Sampling and Labelling Analysis

Samples were obtained from the various markets based on the following inclusion and exclusion criteria.

Inclusion criteria are as follows:

• The sample should have a label and be identified as “tomato paste”.
• Batch number should be available.
• Dates of manufacture and expiry available.
• Indication of country of production or packaging.
• Must either be in a tin and/or sachet.

Exclusion criteria are as follows:

• Absence of a label
• Nonavailability of manufactured and expiry dates
• No inscription of country of origin

The criteria were based on the Ghana FDA requirement for labelling of products except for the presence or absence of the FDA registration number [20]. A total of eight brands, based on availability at the said markets which met the aforementioned criteria, were selected. Five batches (replicates) of each brand were purchased from the markets and assigned a unique identification code (Table 1). A total of 40 samples were obtained for analysis (Table 1). Labels of samples were evaluated to assess conformance to the recommended label content and format as required by regulation [20]. Samples were stored in a cool, dry storage cupboard away from light.

### 2.4. Physical Evaluation

The protocol described by Tsimidou and Boskou [21] was employed to assess the physical and organoleptic attributes of samples; presence of molds or microbial growth, seeds, colour, taste, and appearance were examined and described accordingly.

### 2.5. Determination of Starch Content

#### 2.5.1. Qualitative Tests

The presence of starch in the samples was qualitatively evaluated by light microscopy [22]. About 2 mg of the tomato paste samples was weighed onto a slide, and one drop of 5% iodine solution was added. It was then overlaid with a cover slip and examined for starch grains using a Leica light microscope DM 700 fitted with a camera ICC50 HD (Leica Microsystems, Germany). Photomicrographs of the starch grains, stained blue-black, were taken at ×10 and ×40 magnifications.

#### 2.5.2. Quantitative Test

The total starch content of the tomato paste samples was evaluated as previously described [23, 24] and adopted by the association of official analytical chemists (AOAC, Method 996.11) with slight modification. The method is based on the sequential hydrolysis of starch, first with *α*-amylase and then amyloglucosidase, to D-glucose. D-glucose is then oxidized by reaction with GOPOD reagent to form a dye, quinoneimine, which is measured spectrophotometrically at 510 nm.

To 100 mg of tomato paste in a test tube, 0.2 mL of aqueous ethanol 80% (*v*/*v*) was added to aid dispersion. Thermostable *α*-amylase solution (3.0 mL) buffered at pH 7 in sodium acetate buffer (100 mM) was added to the slurry. The mixture was stirred vigorously in vortex mixer for 10 s and then heated in a water bath at 100°C for 6 min. The test tube was then transferred to another water bath at 50°C, and after approximately 2 min, diluted pullulanase/*β*-amylase solution (4.0 mL) in acetate buffer (pH 5) at room temperature was added with a vigorous stirring on a vortex mixer for 10 s. Incubation was continued at 50°C for 1 h and stirred occasionally.

The content of the test tubes was allowed to cool and transferred quantitatively into a 100-mL volumetric flask and then topped with distilled water to the 100 mL mark. The content was then mixed thoroughly and centrifuged at 3000 rpm for 10 min. The supernatant obtained after the centrifugation was mixed with amyloglucosidase and incubated at 50°C for 10 min. GOPOD reagent (3 mL) was added to the aliquot in the test tube, after which it was incubated at 50°C for 20 min. The same procedure was carried out on 0.1 mL of D-glucose standard solution (1 mg/mL), for the control experiment. The absorbance for each sample was read at 510 nm against the reagent blank from the spectrophotometer.

Total starch content (%) was calculated using the following equation:

(1) $$\begin{array}{c} \text{Total starch content}=A\times F\times 100\times \frac{1}{1000}\times \frac{100}{W}\times \frac{162}{180} \end{array}$$

where *A* is the absorbance of reaction solutions read against reagent blank; *F* is the factor to convert absorbance values to milligram glucose =100 *μ*g glucose/absorbance value for 100 *μ*g glucose; 1000 is the volume correction, that is, 0.1 mL taken from 100 mL; 1/1000 is the conversion from micrograms to milligrams; 100/W is the conversion to 100 mg sample; and 162/180 is the factor to convert from free glucose, as determined, to anhydroglucose, as occurs in starch.

### 2.6. High-Performance Liquid Chromatography (HPLC) Detection of Erythrosine

The presence of erythrosine was determined using a slightly modified validated method described by Vlase et al. [25]. About 5 g of tomato paste products was each weighed into separate conical flasks, and 25 mL of methanol was added. The mixture was placed in a mechanical shaker for 30 min and filtered, using a membrane filter (0.45 *μ*m), into a test tube. The filtrate was transferred into a clean vial, which was loaded onto a preconditioned HPLC machine. Reference erythrosine sample was prepared in methanol at a concentration of 0.01% *w*/*v*. An Agilent (Model No. 1260, Agilent Technologies Inc., Santa Clara, United States) coupled to UV-DAD detector with a binary pump, autosampler, degasser, and a variable wavelength detector constituted the HPLC system. About 0.1 M ammonium acetate buffer (pH = 4) and acetonitrile in the ratio 75: 25, injected at 10 *μ*L at a flow rate of 1 mL/min, were used as the mobile phase. Zorbax SB C18 column 150 × 4.6 mm, 5 *μ*m (Agilent Technologies, United States) was employed as the stationary phase. Detection of erythrosine was done at a wavelength of 527 nm. All data were acquired using the ChemStation software Version Rev B.04.03 (16).

### 2.7. Physicochemical Assessment

#### 2.7.1. Determination of Total Solids

The total solid content was measured by a gravimetric technique [26, 27]. An amount of 5 mg of each sample was weighed into a preweighed crucible. The crucible and its contents were heated for 3 h in an oven at 105°C and thereafter reweighed. The total natural solid (TS) present was determined using the equation below:

(2) $$\begin{array}{c} \text{Total solids }\left(\%\right)=\frac{E}{A}\times 100 \end{array}$$

where *A* is the weight of tomato paste and *E* is the weight of tomato paste after drying.

#### 2.7.2. Determination of pH

The pH of the tomato samples was determined using a Mettler Toledo pH meter at 20°C [26]. Samples were diluted with deionized water in a ratio of 1:1 and vortexed for 30 s to obtain a homogeneous blend. The probe of the pH meter was then inserted, and the values were read.

#### 2.7.3. Determination of Titratable Acidity (TA)

The TA of samples was determined with a Mettler Toledo pH meter by mixing tomato paste samples with deionized water in a ratio of 1:5 and titrating with 0.1 N NaOH until a pH of 8.2 was reached. The TA results were calculated using the equation below and reported as percent citric acid/kilogram tomato paste [26].

(3) $$\begin{array}{c} \text{TA }\left(\%\right)=\text{Vol}\times 0.1\times 100\text{g}\times \frac{0.064}{M} \end{array}$$

where Vol is the volume of NaOH used, 0.064 is the conversion factor, and *M* is the mass of the sample.

#### 2.7.4. Determination of TSSs

The TSS measurements were carried out by diluting samples with deionized water in a ratio of 1:5. The mixtures were vortexed for a minute for homogeneity and filtered to obtain the serum. About 1 mL of serum from each sample was pipetted into the prism of a temperature-controlled refractometer and the TSS, and the temperature was recorded [26].

#### 2.7.5. Determination of Colour of Tomato Paste

The colour was determined using the CIELAB colour system described by the GSA [26]. The colour parameters were measured using a Konica Minolta brand chromameter, Model CR_400. The CIELAB coordinates were measured and expressed in terms of *L*^∗^ (*L*^∗^ = 0 for black and *L*^∗^ = 100 for white) and chromaticity parameters *a*^∗^ (green [−] and red [+]) and *b*^∗^ (blue [−] and yellow [+]).

### 2.8. Statistical Analysis

The statistical analysis of the quantitative data obtained was conducted using GraphPad Prism® Ver. 8 (GraphPad Software, Inc., La Jolla, CA, United States). Mean values of groups (*n* = 5) were compared by one-way ANOVA, followed by Dunnett's test for multiple comparison. Values were considered significant at 95% confidence level.

## 3. Results

### 3.1. Label Analysis

All the test samples had the manufacturer or distributor details, batch numbers, net weights, and best before dates clearly stated. However, three products, C, D, and G, failed the quantitative ingredient declaration (QUID) (Table 2).

### 3.2. Physical Evaluation

The physical attributes of each tomato paste product were observed and documented (Table 3). All products had a characteristic taste, with no visible signs suggestive of microbial growth or specks of seeds in the products.

### 3.3. Starch Content

#### 3.3.1. Qualitative Analysis

**Figure 2:**
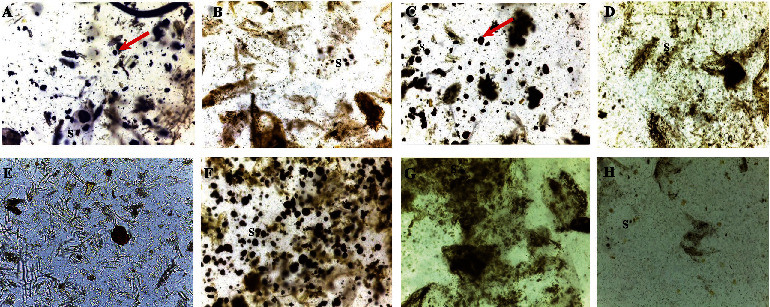
Micrographs of tomato paste products mounted with N/50 iodine.

Qualitative microscopy revealed the starch grains as blue-to-black spots (Figure 2). Abundant starch grains were found in Samples A, C, and F, whereas the remaining samples had very few grains interspersed with some fibres (Figure 2).

#### 3.3.2. Quantitative Analysis of Starch

**Figure 3:**
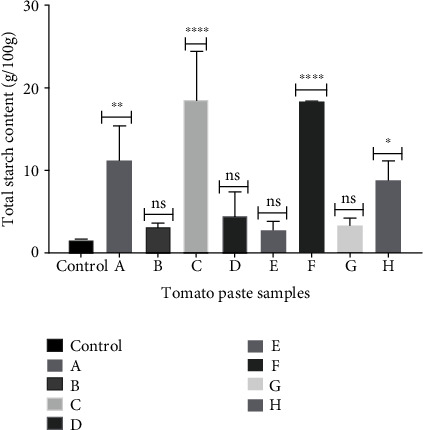
Total starch content of market samples of tomato paste compared to control.

In the total starch content analysis, the paste (puree) prepared from fresh tomatoes and used as control recorded a starch content of 1.55 ± 0.095 g/100 g. This was followed by Samples E, B, G, and D, respectively, all recording starch contents of not more than 4.5 g/100 g (Figure 3). The highest amount of starch, in excess of 18 g/100 g, occurred in Samples F and C, with A and H following closely (Figure 3). The starch content for A, C, and F was significantly (*p* < 0.05) higher than the other sampled products. This agreed with the qualitative microscopy starch analysis (Figure 2), which displayed the considerable adulteration of these products (Samples F, C, and A) with copious amounts of starch.

### 3.4. Colour of Tomato Paste Samples

One characteristic parameter assessed for tomato paste is the colour. A characteristic red colour is well-known and accepted for tomato paste.  Table 4 provides the instrumental colour profile of the samples assessed with a chromameter, presented as the *a*/*b* ratio.

### 3.5. Artificial Colourant Erythrosine as Adulterant

**Figure 4:**
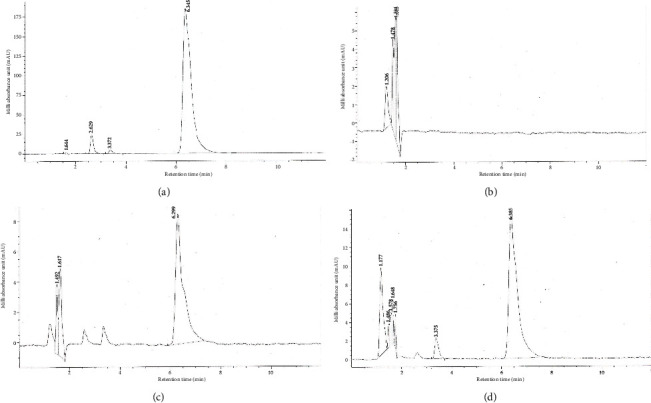
HPLC chromatogram of (a) erythrosine, (b) home-made puree as control, and samples (c) A and (d) F.

A validated HPLC method afforded the elution of the reference colourant erythrosine at an average retention time of 6.382 min (Figure 4(a)). The home-made tomato puree did not reveal the presence of erythrosine (Figure 4(b)). The synthetic red dye, however, was eluted from two out of the eight test samples (see Figures S1–S8 in Supporting Information). The dye was eluted from all five replicates of both of A and F, with average retention times of 6.293 ± 0.007 and 6.381 ± 0.012 min, respectively (Figures 4(c) and 4(d)).

### 3.6. Physicochemical Characteristics of Products

#### 3.6.1. Total Solids, pH, and TA of Products

The TS content of a tomato product provides its characteristic name, be it ketchup, puree, paste, or concentrated paste. Tomato paste products are required to have a TS concentration between 24% and 44% for it to be labelled a tomato paste Mizutani [13]. From the results obtained (Table 5), 50% of the products had a TS above the minimum requirement to be labelled tomato paste, the highest being the sample labelled H, with a TS of 30.5%.

In the test for acidity or alkalinity, all the samples had a pH < 4.60 (Table 5) and thus were within the pH value acceptable for a tomato product. A similar factor that predicts the impact of pH (from organic acids) on the flavour of products is TA. The TA in tomato and tomato products is expressed as the percentage of citric acid. The TA values of the tomato paste samples ranged between 0.848% and 1.739% citric acid (Table 5).

#### 3.6.2. TSS

The TSS indicates the amount of sugars and other dissolved solids present in a given sample. It is expressed as °Brix.  Table 4 shows a TSS content range from 5.73 to 10.60 °Brix, with Sample D having the highest and E the lowest (Table 6).

### 3.7. Correlation Analysis Between Parameters

A simple correlation analysis between the measured parameters revealed several strong positive correlations and a few weak ones (Table 7).

## 4. Discussion

### 4.1. Product Information and Label Analysis

The minimum quality requirements and ingredients that can be used in tomato concentrates are defined in local and international legislations and standards to protect consumer health and ensure fair food trade [28]. Despite these regulatory requirements, issues of adulteration continue to be on the rise, typically to produce products that are more appealing to consumers at low production costs for increased profit [7]. This was evident in the current research, which assessed brands of tomato paste in the Ghanaian markets for conformity to regulatory standards.

All the tomato samples were labelled in terms of their place of origin, manufacturer or distributor details, batch numbers, net weights, and best before dates (Table 2). However, Products C, D, and G failed the QUID, and this is of great significance since most consumers depend on it to make informed product purchases [29]. For traceability purposes, labelling is necessary for packaged foods [30]. The QUID also enables consumers to make informed purchases of healthier foods, facilitates product quality comparisons, prevents adulteration, and encourages the development of higher quality food products [31]. Foreign or imported products constituted 62.5% of the sampled tomato concentrates. This corroborates with records that the majority of the 100,000 metric tons of tomato paste consumed annually in Ghana comes from imports [32, 33].

### 4.2. Physical Attributes and Detection of Colourant Erythrosine

In the physical attribute assessment, none of the products had any bad odour, mold, seeds, or any parameter suggestive of product deterioration. Three of the samples had a deep-red colour, with the rest being bright red (Table 3). The colour of tomato paste has been reported to be the main quality specification, according to US and Canadian standards [34], and the *a*/*b* ratio has been used as a specification quality for the product. According to Turkish and Egyptian standards, the *a*/*b* ratio should not be less than 1.8 and any value less than this is not of good quality [34, 35]. Comparing the instrumental colour with the organoleptic characteristics (Tables 3 and 4), Samples B, D, and G were described to have a dark-red colour. However, B and D had *a*/*b* ratios of 2.48 and 2.51, respectively, which were above the set standard. Sample G, with the lowest *a*/*b* ratio of 1.69, is therefore below standard and should be redrawn from the market and reconstituted. All other samples were between 2.17 and 2.51 and thus were of good quality within acceptable set standards.

The red colour of tomatoes is attributed to carotenoids, of which lycopene constitutes approximately 80% [7]. The food industry uses food colourants to meet the expectations of consumers, who commonly associate colour with product quality. A synthetic dye commonly used to enhance the colour of tomato products is erythrosine. HPLC analysis did not reveal the presence of erythrosine in the home-made puree or in most of the products (75%). The synthetic dye, however, was detected in Samples A and F (Figure 4). Erythrosine (2,4,5,7-tetraiodofluorescein) is a cherry-pink food colourant with a poly-iodinated xanthene structure (Figure 1). It is widely used in cosmetics, drugs, and food [12, 14, 36]. Although its utilization at low concentrations is permitted by the US FDA, cumulative evidence of toxicity pointing to studies suggesting it causes carcinogenicity, hyperactivity, and other neurobehavioral effects in children has resulted in calls by consumer groups to ban the colourant [10]. As a result of this, food regulatory bodies have made laws to control the use of this colourant. Recently, the Ghana FDA banned 16 tomato paste products on the market due to the presence of starch and erythrosine, which was not indicated on the label [20]. This contravenes the general labelling law, LI 1541. Again, the addition of this colourant, according to the FDA, is in contravention to Ghana Standard for Tomato Paste [26]. Therefore, Samples A and F should be withdrawn from the market.

### 4.3. Physicochemical Quality of the Products

Besides the colourant, other physicochemical assessments were conducted on the products to determine certain parameters, such as the TA and pH. These are important quality parameters for tomatoes and their products. For tomato paste, a low pH is desirable as it inhibits putrefying microbes (bacteria and fungi). Tomato pastes and mixes should have a pH no higher than 4.6 (pH < 4.6), according to GSA [26]. A low pH assures the product's safety as it is critical to controlling the growth of spoilage and harmful microorganisms. All samples evaluated had a pH less than the standard value, with the highest being Sample D with 4.18 and the lowest being Sample H with 3.74. The TA, on the other hand, gives an indication of how much of the organic acids present in a food product affect the pH and flavour. It also helps us to identify how much of a particular organic acid is present in a product. The TA of tomato and its products are expressed in percent citric acid; thus, citric acid is the organic acid of interest here, even though other organic acids are present. Sample E recorded the lowest TA value of 0.848% citric acid/100 g tomato paste, whilst Sample G recorded the highest, with 1.760% citric acid/100 g tomato paste. The characteristic taste and odour of tomato pastes and mixes are derived from citric acid, which is naturally abundant in tomatoes and important for lowering the pH for product safety [37]. However, the maturity level and handling and processing conditions all influence the levels found in the final product, as citric acid easily degrades. Citric acid is therefore added to such products as an acidity regulator to control the pH of the products. Aykas, Rodrigues Borba, and Rodriguez-Saona [38] reported a TA range between 1.0 and 1.9 for different tomato paste samples when assessing the quality of tomatoes using portable mid-infrared spectroscopy. Devseren et al. [34] also recorded a range of 0.727–2.318 when the quality of tomato pastes processed at different temperatures and processing times was assessed. These ranges corroborate with those recorded in the present studies (Table 3).

The TS concentration obtained from the samples ranged from 18.06% to 30.05%. A TS between 9% and 12% denotes a tomato puree, depending on the extraction method, with cold extraction being the lowest (9%) and hot extraction methods being the highest (12%). It should be noted that tomato sauce (TS between 24% and 25%) and ketchup (TS between 28% and 30%) are different from tomato pastes (24% and 44%) [8, 39], which require all additives to be listed on the label. The different TS concentrations draw the lines between the different tomato products, such as purees, sauces, and ketchup. Tomato mixes (which contain other ingredients), according to standards set by the Ghana Standards Authority, can have a total solid concentration of at least 20%. Fifty percent of the samples did not meet the criteria to be labelled as tomato paste since they had a TS of less than 24% (Table 5). The product with the least TS concentration, Sample E with 18.06%, is below the standards set for both tomato pastes and mixes and hence does not fall under any of these categories. A tomato-based product with a TS content above 33% is described as a concentrated tomato paste. None of the products were within this category.

In the TSS assessment (Table 6), measured by the Brix content, all the samples ranged between 5.73 and 10.60 at a temperature range of 27.5°C–28.46°C. The TSS for tomato paste is a minimum of 7%, according to the requirement of the GSA. Sample D recorded the highest, with 10.60, whereas Sample E had the lowest, with 5.73. The Brix content gives an idea about the quantity of sugar, mostly natural sugars (sucrose, fructose), in the tomato paste samples. The higher the Brix content, the higher the sugar content of the tomato paste. Thus, Products B, D, F, and H, with Brix contents > 9.0, may contain considerable amounts of sugar. Sugars and starch are not permitted in tomato paste, according to regulatory requirements [17]. All the products contained some amount of starch, but A, C, and F had contents above 10 g/100 g of the product. This deliberate adulteration with copious amounts of bulking agent calls for a heightened regulatory scrutiny of these products to safeguard the health of consumers. Periodic postmarket surveillance by the regulatory authority will identify such substandard and adulterated products on the market.

The correlation analysis showed 17 positive correlations between parameters relative to negative ones (4). The negative correlations were all weak. The strong positive correlation between the two parameters (refractive index/°Brix *r* = 0.9606) is not surprising since the dissolved solids, measured by °Brix, contribute to the light scattering properties of the samples. This implies that changes in refractive index will be mirrored by changes in °Brix and vice versa, suggesting a possible interchangeability for quality control purposes. The apparent influence of TAs on the refractive index was also evident, although not as pronounced (*r* = 0.7272). Interestingly, the TA did not correlate negatively with pH as expected. This suggests the influence of other properties or substances on the pH of the tomato paste samples tested. This will require further research with a larger sample size and the use of other chemometric strategies.

## 5. Conclusions

Eight tomato paste products were sampled during the survey, all of which contained some amount of starch, with three of them adulterated with copious amounts of the thickening agent although. Additionally, two products contained the erythrosine colourant, and some products did not conform to physicochemical parameters consistent with regulatory standards for tomato pastes. This calls for heightened regulatory scrutiny and frequent postmarket surveillance to safeguard the health of consumers. In addition to the qualitative determination of erythrosine, a validated HPLC method will have to be developed for the routine quantitative analysis of the dye. Future research should include all the different types of processed tomato products on the market, with a large sample size. This will enable the use of chemometrics to enable the in silico prediction of adulteration.

## Figures and Tables

**Table 1:** Selected brands of tomato paste and assigned identification codes.

**Test samples**	**Assigned code**	**Replicates**
1	A	A1, A2, A3, A4, A5
2	B	B1, B2, B3, B4, B5
3	C	C1, C2, C3, C4, C5
4	D	D1, D2, D3, D4, D5
5	E	E1, E2, E3, E4, E5
6	F	F1, F2, F3, F4, F5
7	G	G1, G2, G3, G4, G5
8	H	H1, H2, H3, H4, H5

*Note:* Codes A–H represent the various brands of tomato pastes obtained from the market. The designation “1–5” represents the five replicates.

**Table 2:** Label analysis of tomato paste products.

**Product code**	**Manufacturer's and distributor's information**	**Country of origin**	**Batch number**	**Quantitative ingredient declaration**	**Net weight**	**Best before dates**	**Designation**
A	+	+	+	+	+	+	Local
B	+	+	+	+	+	+	Foreign
C	+	+	+	−	+	+	Local
D	+	+	+	−	+	+	Foreign
E	+	+	+	+	+	+	Foreign
F	+	+	+	+	+	+	Local
G	+	+	+	−	+	+	Foreign
H	+	+	+	+	+	+	Foreign

*Note:* A–H; tomato paste products sampled from the market. Plus (+) sign denotes present while minus (−) denotes absent.

**Table 3:** Physical attributes of tomato paste products.

**Test samples**	**Physical attributes**			
	**Taste**	**Colour**	**Appearance**	**Seeds/molds**
A	Characteristic	Bright red	Paste	Absent
B	Characteristic	Dark red	Paste	Absent
C	Characteristic	Bright red	Paste	Absent
D	Characteristic	Dark red	Paste	Absent
E	Characteristic	Bright red	Paste	Absent
F	Characteristic	Bright red	Paste	Absent
G	Characteristic	Dark red	Paste	Absent
H	Characteristic	Bright red	Paste	Absent

*Note:* Codes A–H represent the various brands of tomato pastes obtained from the market.

**Table 4:** Colour of tomato paste samples presented as *a*/*b* ratio.

**Sample code**	**a**/**b**
A	2.20 ± 0.0078
B	2.48 ± 0.0021
C	1.99 ± 0.0024
D	2.51 ± 0.0002
E	2.18 ± 0.0038
F	2.17 ± 0.0011
G	1.69 ± 0.0003
H	2.43 ± 0.0009

*Note:* A–H; tomato paste products sampled from the market.

**Table 5:** Total solid, pH, and titratable acidity of tomato paste samples.

**Sample code**	**TS (%)**	**pH**	**TA (% citric acid/100 g tomato paste)**
A	23.25 ± 0.0146	3.87 ± 0.0007	0.981 ± 0.0680
B	23.53 ± 0.0083	4.06 ± 0.0022	1.739 ± 0.0512
C	26.91 ± 0.0004	3.77 ± 0.0003	1.288 ± 0.0501
D	27.44 ± 0.0280	4.18 ± 0.0003	1.546 ± 0.0348
E	18.06 ± 0.0876	3.81 ± 0.0001	0.848 ± 0.0461
F	28.44 ± 0.0641	3.94 ± 0.0001	1.120 ± 0.0021
G	21.00 ± 0.0545	4.05 ± 0.0001	1.760 ± 0.0250
H	30.05 ± 0.0082	3.74 ± 0.0001	1.408 ± 0.0512

*Note:* A–H; tomato paste products sampled from the market.

Abbreviations: TA = titratable acidity; TS = total solids.

**Table 6:** Total soluble solids of tomato paste products.

**Sample code**	**°Brix**	**Refractive index**	**Temperature (°C)**
A	6.68 ± 0.27	1.342	27.5
B	10.08 ± 0.21	1.348	28.07
C	7.43 ± 0.13	1.345	28.40
D	10.60 ± 0.33	1.348	27.98
E	5.73 ± 0.05	1.341	28.00
F	9.62 ± 0.45	1.347	27.98
G	8.35 ± 0.26	1.345	28.00
H	9.80 ± 0.33	1.346	28.18

*Note:* A–H; tomato paste products sampled from the market.

**Table 7:** Correlation matrix of the determined parameters of the tomato paste samples.

	**TS (%)**	**pH**	**TA**	**°Brix**	**Refractive index**	**Temperature (°C)**	**Total starch**
TS (%)	1	−0.0796	0.1822	0.6774	0.6487	0.3257	0.5728
pH	−0.0796	1	0.6087	0.5579	0.5694	−0.2299	−0.4593
TA	0.1822	0.6087	1	0.6929	0.7272	0.3329	−0.3777
**°**Brix	0.6774	0.5579	0.6929	1	0.9606	0.2419	−0.0579
Refractive index	0.6487	0.5695	0.7272	0.9605	1	0.3826	0.0483
Temperature (°C)	0.3257	−0.2299	0.3329	0.2419	0.3826	1	0.1883

## Data Availability

The data presented in this study are available in the article and Supporting Information.

## Supporting Information

Supporting Information Additional supporting information can be found online in the Supporting Information section. The HPLC chromatograms of all samples are available in Supporting Information. Supporting Information S1–S8 show the chromatographs of the Tomato Samples A–H, respectively, indicating whether erythrosine was present or not. Supporting Information S9 shows the chromatograph for the reference erythrosine sample.
