# Supplementary figures and images for: Quality Assessment of Tomato Paste Products on the Ghanaian Market: An Insight Into Their Possible Adulteration
Source: Int J Food Sci. 2024 Sep 9;2024:8285434. doi: 10.1155/2024/8285434 (PMC11405106; doi:10.1155/2024/8285434)

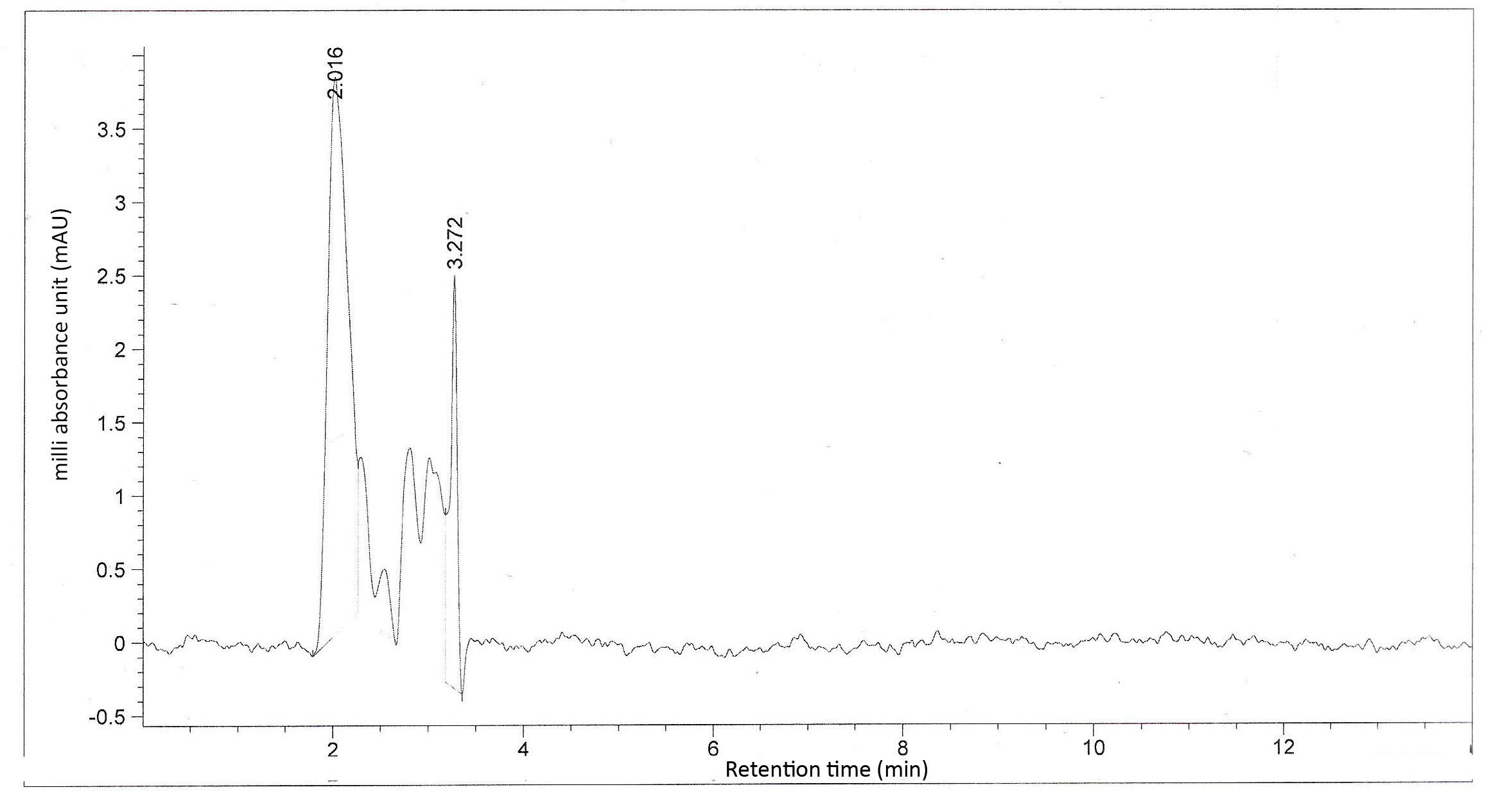

Supplement: Supporting Information — Additional supporting information can be found online in the Supporting Information section. The HPLC chromatograms of all samples are available in Supporting Information. Supporting Information S1–S8 show the chromatographs of the Tomato Samples A–H, respectively, indicating whether erythrosine was present or not. Supporting Information S9 shows the chromatograph for the reference erythrosine sample. [file 8285434.f1.zip › Boakye et al.,_Supplementary material 8(Sample H- Erythrosine Absent) (1).png]

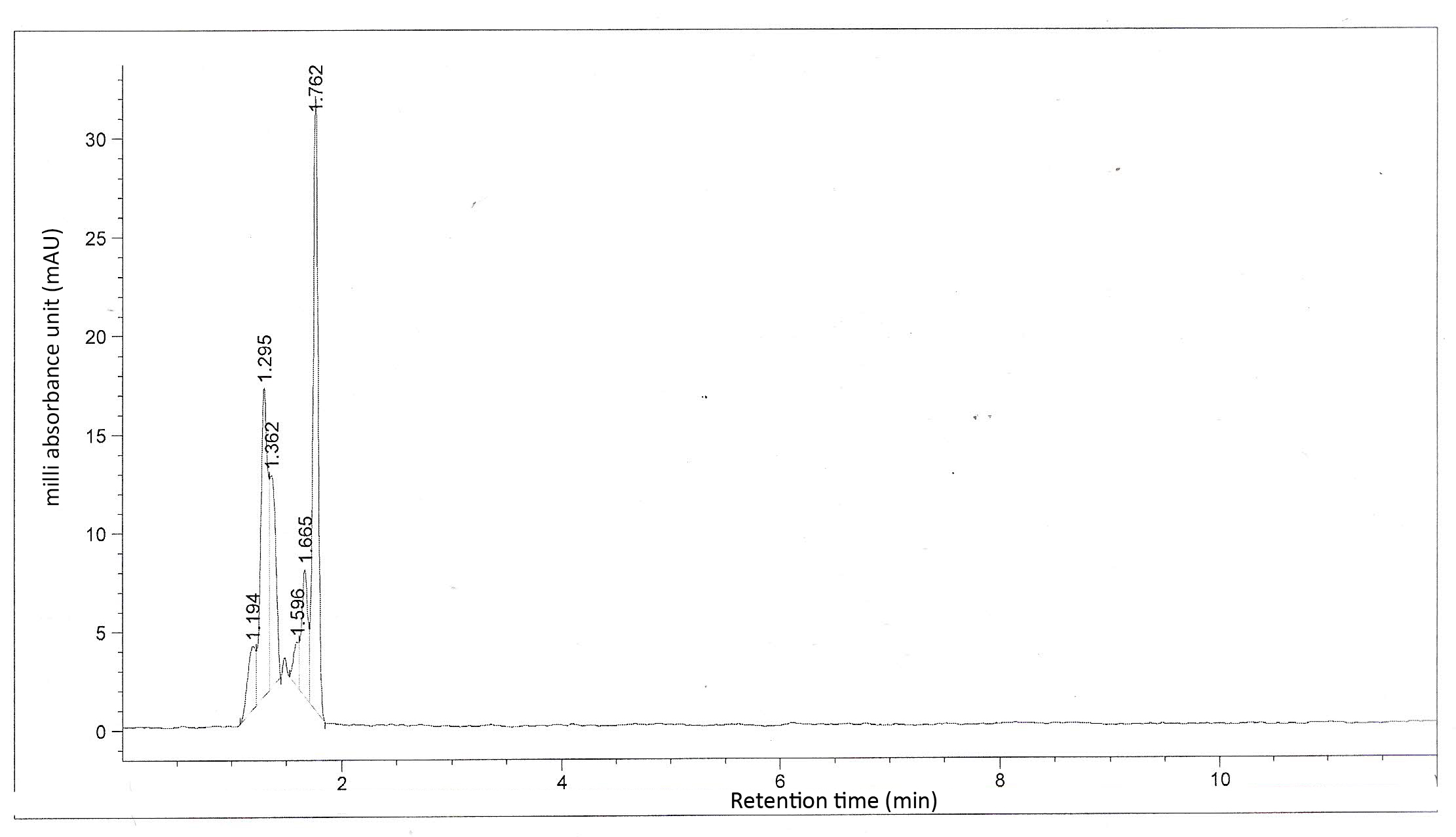

Supplement: Supporting Information — Additional supporting information can be found online in the Supporting Information section. The HPLC chromatograms of all samples are available in Supporting Information. Supporting Information S1–S8 show the chromatographs of the Tomato Samples A–H, respectively, indicating whether erythrosine was present or not. Supporting Information S9 shows the chromatograph for the reference erythrosine sample. [file 8285434.f1.zip › Boakye et al._ Supplementary material 2 (Sample B - Erythrosine Absent) (1).png]

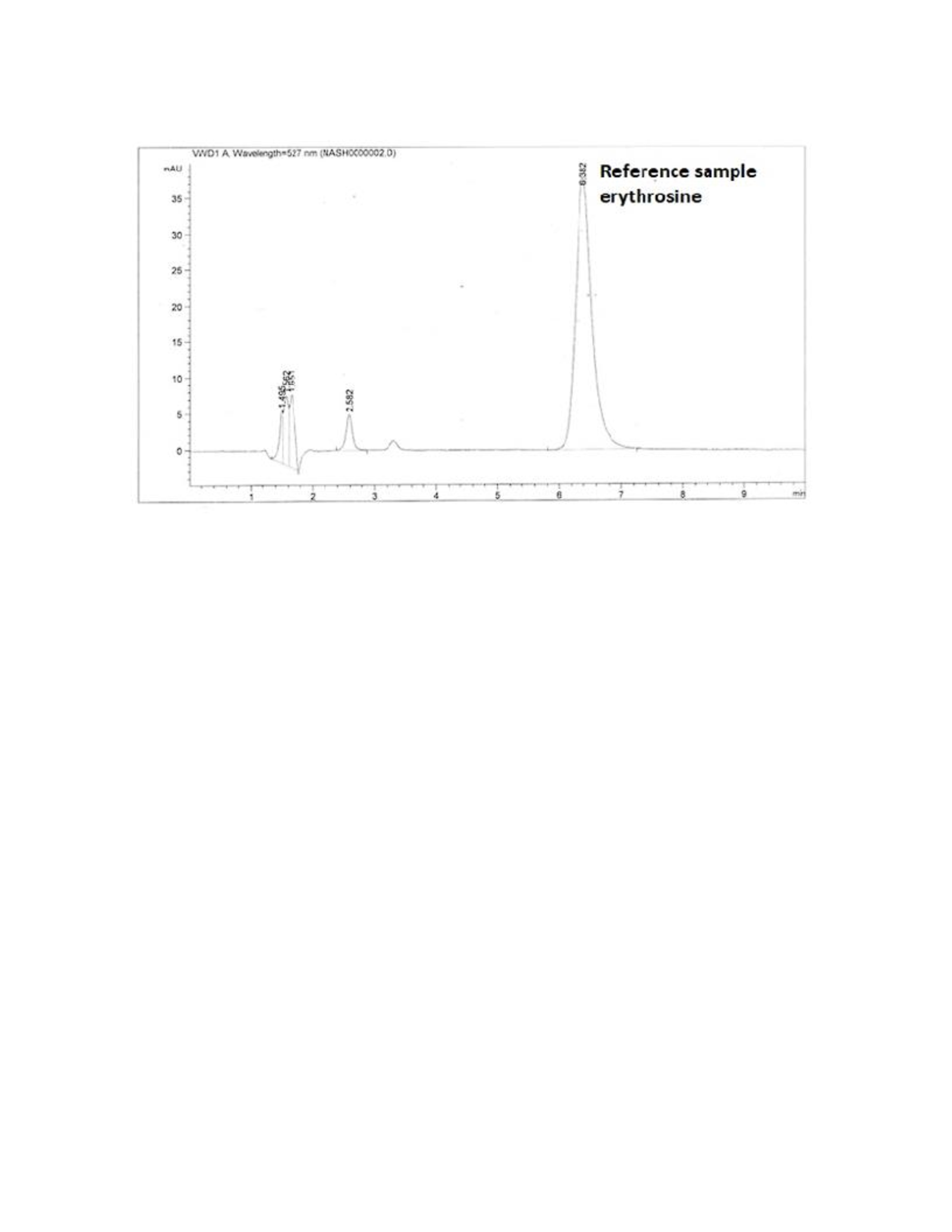

Supplement: Supporting Information — Additional supporting information can be found online in the Supporting Information section. The HPLC chromatograms of all samples are available in Supporting Information. Supporting Information S1–S8 show the chromatographs of the Tomato Samples A–H, respectively, indicating whether erythrosine was present or not. Supporting Information S9 shows the chromatograph for the reference erythrosine sample. [file 8285434.f1.zip › Boakye et al._ Supplementary material 9(Reference Chromatograph for Erythrosine) (1).png]

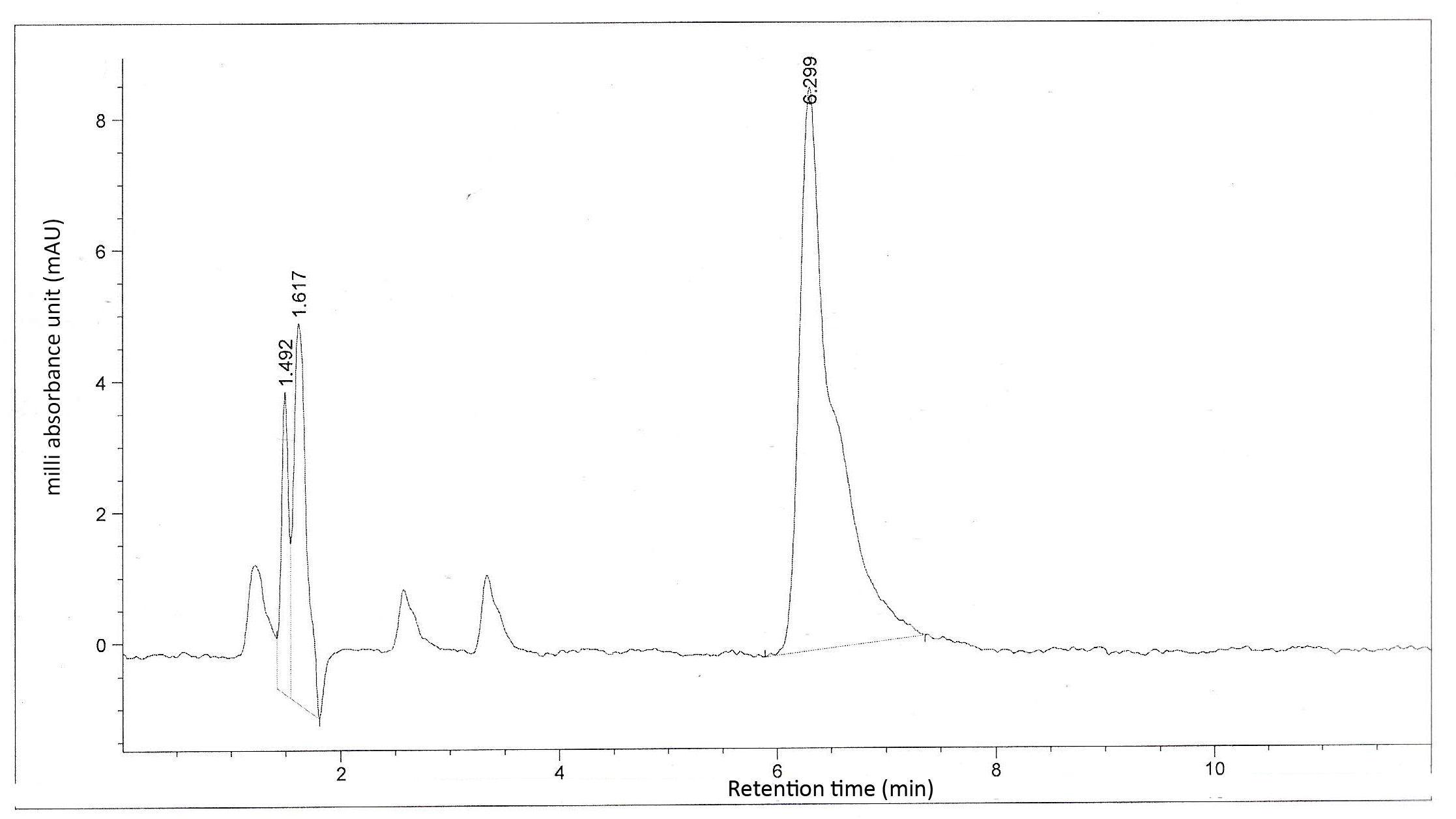

Supplement: Supporting Information — Additional supporting information can be found online in the Supporting Information section. The HPLC chromatograms of all samples are available in Supporting Information. Supporting Information S1–S8 show the chromatographs of the Tomato Samples A–H, respectively, indicating whether erythrosine was present or not. Supporting Information S9 shows the chromatograph for the reference erythrosine sample. [file 8285434.f1.zip › Boakye et al._Supplementary material 1 (Sample A - Erythrosine Present) (1).png]

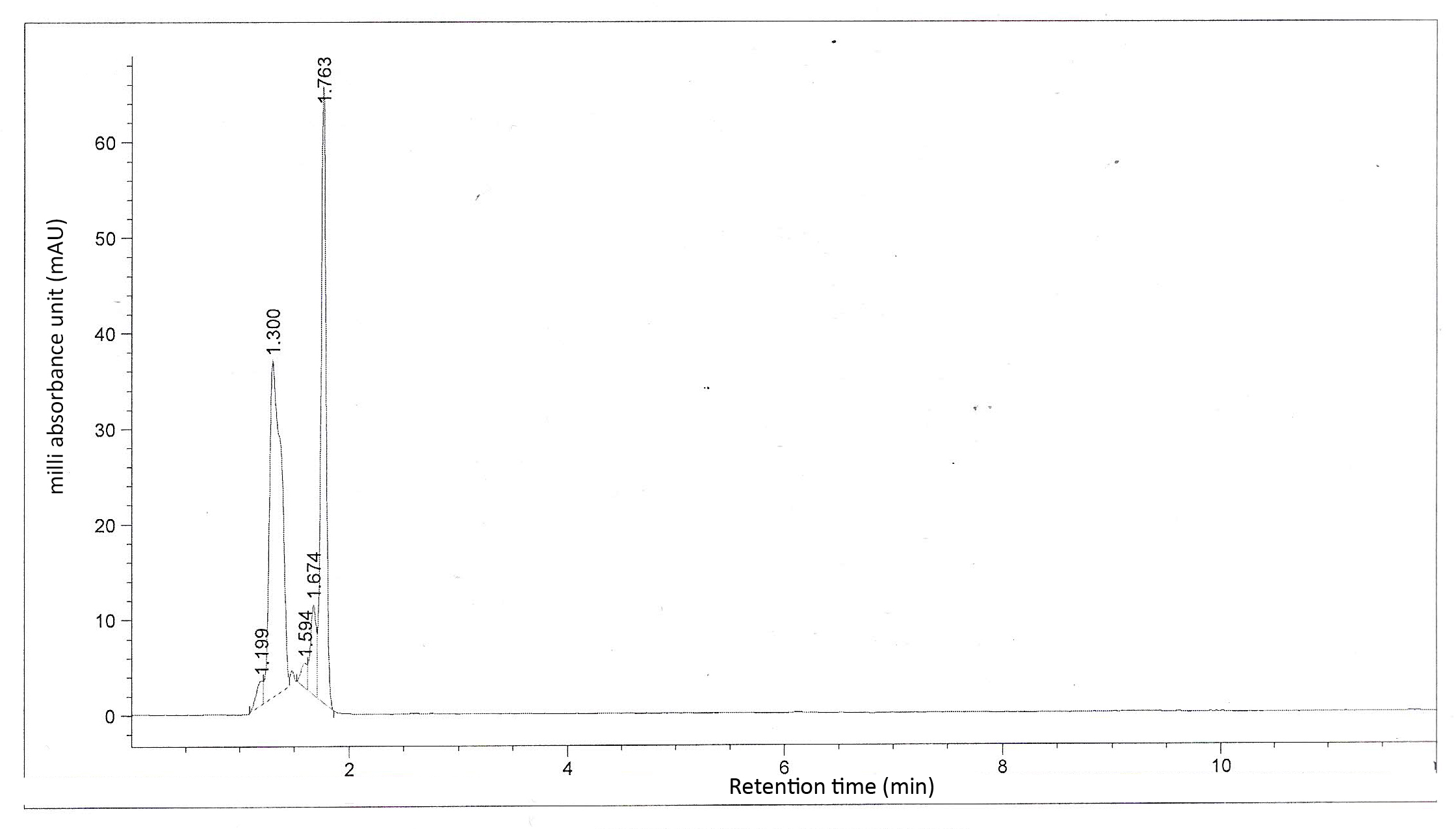

Supplement: Supporting Information — Additional supporting information can be found online in the Supporting Information section. The HPLC chromatograms of all samples are available in Supporting Information. Supporting Information S1–S8 show the chromatographs of the Tomato Samples A–H, respectively, indicating whether erythrosine was present or not. Supporting Information S9 shows the chromatograph for the reference erythrosine sample. [file 8285434.f1.zip › Boakye et al._Supplementary material 3 (Sample C - Erythrosine Absent) (1).png]

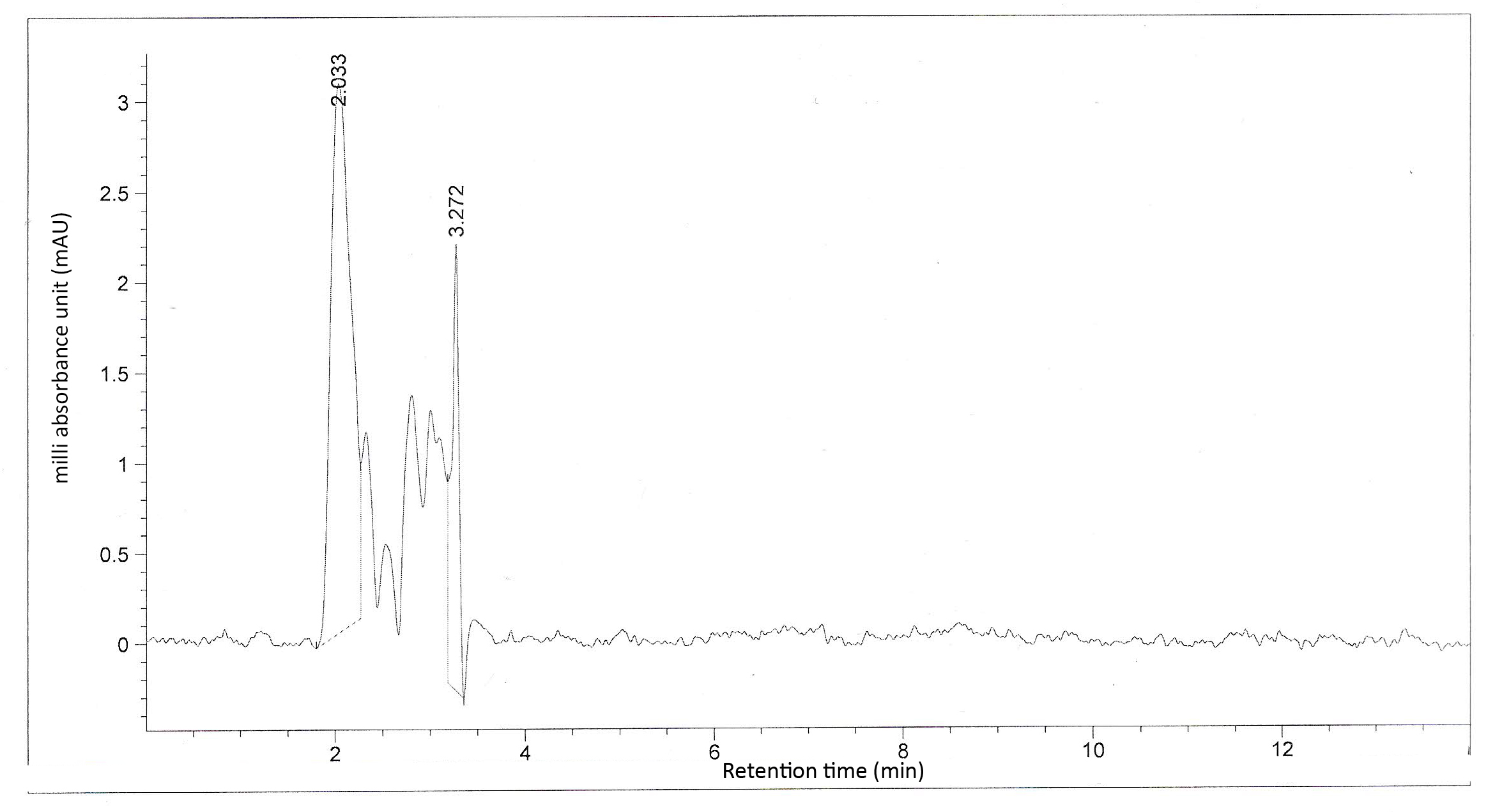

Supplement: Supporting Information — Additional supporting information can be found online in the Supporting Information section. The HPLC chromatograms of all samples are available in Supporting Information. Supporting Information S1–S8 show the chromatographs of the Tomato Samples A–H, respectively, indicating whether erythrosine was present or not. Supporting Information S9 shows the chromatograph for the reference erythrosine sample. [file 8285434.f1.zip › Boakye et al._Supplementary material 4 (Sample D - Erythrosine Absent) (1).png]

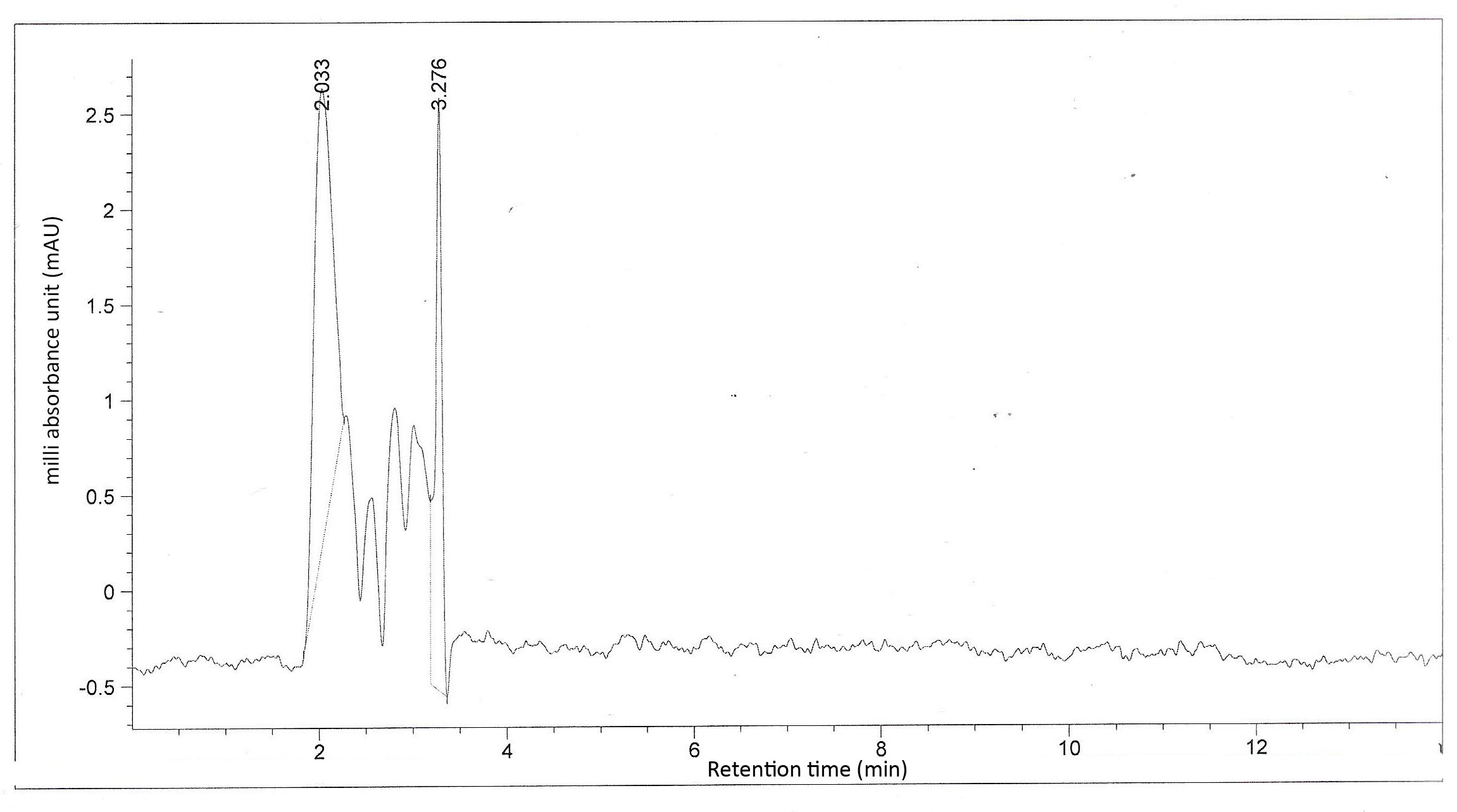

Supplement: Supporting Information — Additional supporting information can be found online in the Supporting Information section. The HPLC chromatograms of all samples are available in Supporting Information. Supporting Information S1–S8 show the chromatographs of the Tomato Samples A–H, respectively, indicating whether erythrosine was present or not. Supporting Information S9 shows the chromatograph for the reference erythrosine sample. [file 8285434.f1.zip › Boakye et al._Supplementary material 5 (Sample E - Erythrosine Absent) (1).png]

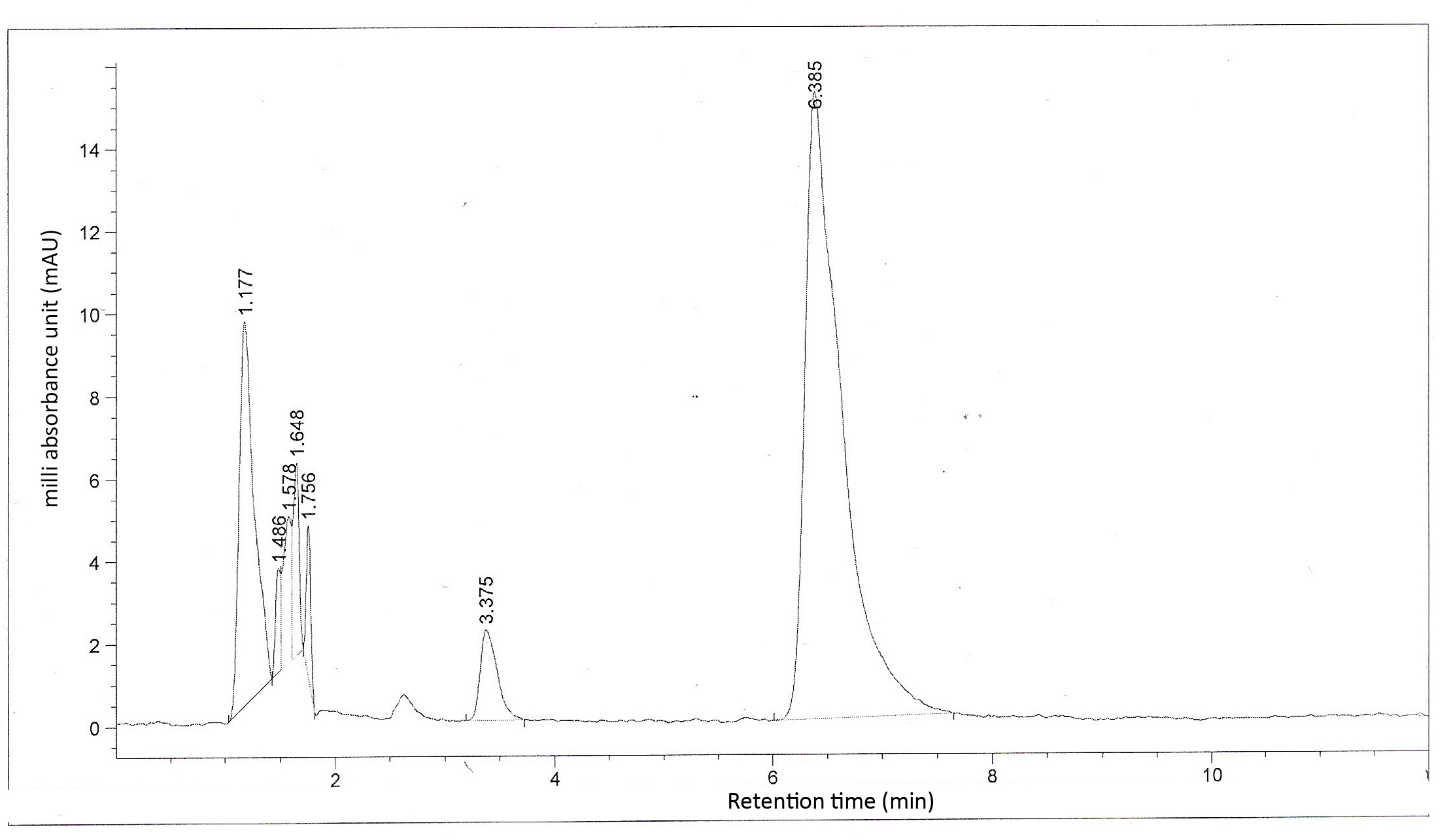

Supplement: Supporting Information — Additional supporting information can be found online in the Supporting Information section. The HPLC chromatograms of all samples are available in Supporting Information. Supporting Information S1–S8 show the chromatographs of the Tomato Samples A–H, respectively, indicating whether erythrosine was present or not. Supporting Information S9 shows the chromatograph for the reference erythrosine sample. [file 8285434.f1.zip › Boakye et al._Supplementary material 6 (Sample F - Erythrosine Present) (1).png]

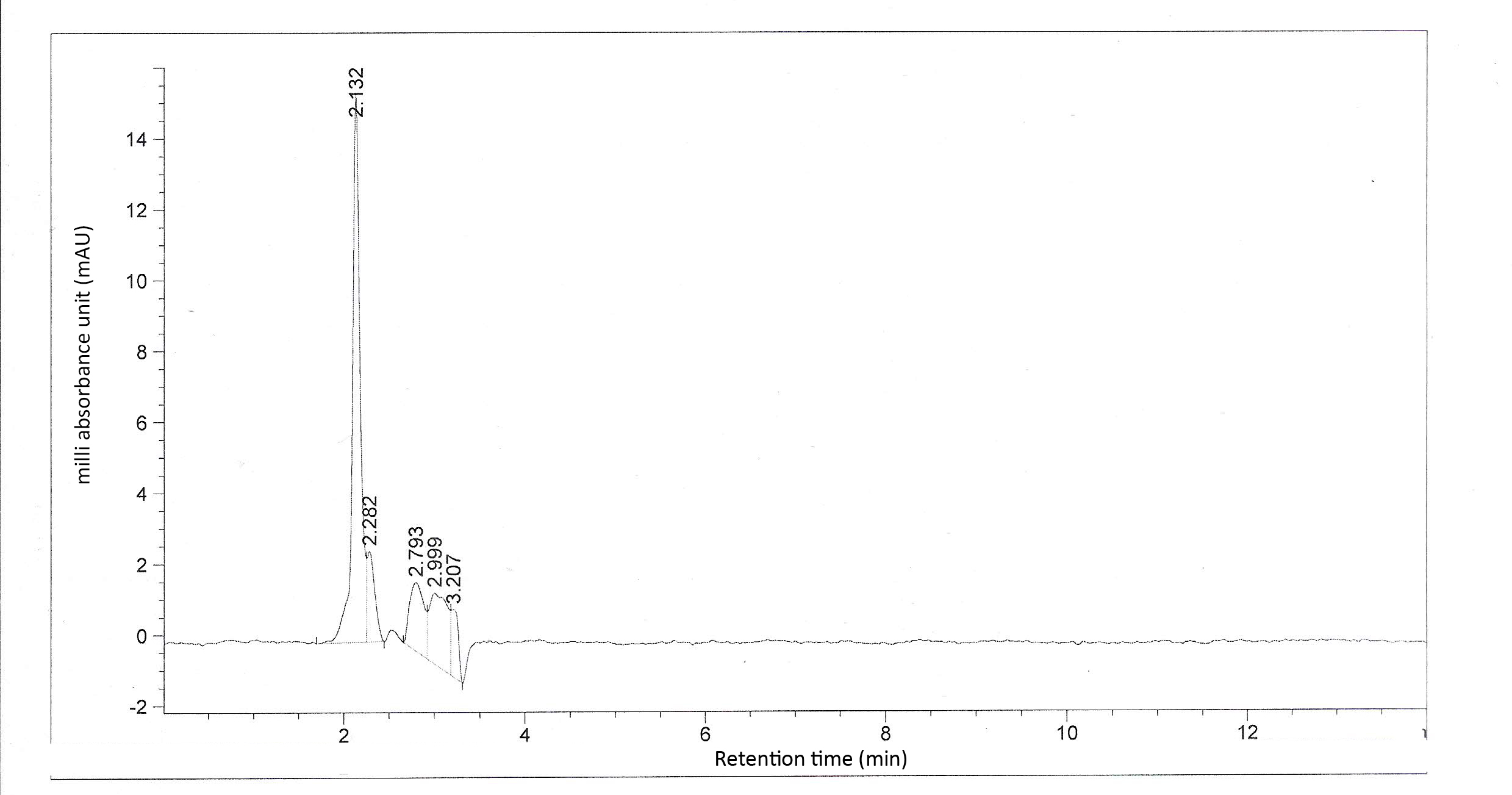

Supplement: Supporting Information — Additional supporting information can be found online in the Supporting Information section. The HPLC chromatograms of all samples are available in Supporting Information. Supporting Information S1–S8 show the chromatographs of the Tomato Samples A–H, respectively, indicating whether erythrosine was present or not. Supporting Information S9 shows the chromatograph for the reference erythrosine sample. [file 8285434.f1.zip › Boakye et al._Supplementary material 7(Sample G - Erythrosine Absent) (1).png]
